# Synthesis, Structure and Antitumour Properties of a New 1,2-Propylenediaminetetraacetate-Ruthenium(III) Compound

**DOI:** 10.1155/MBD.1995.211

**Published:** 1995

**Authors:** R. Vilaplana, M. A. Romero, M. Quirós, J. M. Salas, F. González-Vílchez

**Affiliations:** 1 Dpto. de Quífmica Inorgánica F. de Química Universidad de Sevilla Sevilla 41071 Spain; 2 Dpto. de Quífmica Inorgánica F. de Química Universidad de Granada Granada 18071 Spain

## Abstract

A novel complex formed by ruthenium (III) and the sequestering ligand 1,2-propylenediaminetetraacetic acid (PDTA) has been synthetized and characterized. The structure of the monomeric compound, studied by X-ray diffraction , shows an almost symmetric octahedral geometry around the metal ion, with two chlorine atoms in a *cis* conformation. The antitumour activity against a variety of murine and human cancers is reported.


### SYNTHESIS, STRUCTURE AND ANTITUMOUR PROPERTIES OF A NEW 1,2-PROPYLENEDIAMINETETRAACETATE-RUTHENIUM(III) COMPOUND

R. Vilaplana[1], M.A. Romero[2], M. Quirós[2], J.M. Salas[2] and F. González-Vílchez*[1]

[1] Dpto. de Química Inorgánica, F. de Química, Universidad de Sevilla, 41071 Sevilla, Spain

[2] Dpto. de Química Inorgánica, F. de Ciencias, Universidad de Granada, 18071 Granada, Spain

### Abstract

A novel complex formed by ruthenium (III) and the sequestering ligand 1,2-propylenediaminetetraacetic acid (PDTA) has been synthetized and characterized. The structure of the monomeric compound, studied by X-ray diffraction , shows an almost symmetric octahedral geometry around the metal ion, with two chlorine atoms in a *cis* conformation. The antitumour activity against a variety of murine and human cancers is reported.

### Introduction

Many compounds of transition metals with 1,2-propylenediamine-N,N,N′,N′-tetraacetic acid (PDTA, Figure 1), have been studied in recent years, although those formed by ruthenium have been object of scarce attention[1-4]. Compounds formed by this chelating agent, a methyl derivative of EDTA, are of about two orders of magnitude more stable than the analogs formed with the parent ligand. Furthermore, ruthenium complexes with PDTA may be of interest because of the easy conversion of this element from one oxidation state into another at the appropriate experimental conditions, and because of the tendency of its compounds to undergo hydrolysis. Advantage can be taken from the ready availability of both the Ru(III) and Ru(II) oxidation states under physiological conditions and the general inertness of these ions toward substitution, when coordinated to nitrogen ligands[5]. All the referred properties are in close connection to the ability of the ruthenium complexes to act as potential antitumour drugs[6-9].

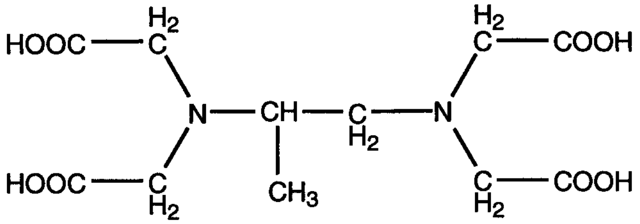

**Figure 1**. Schematic structure of the ligand PDTA

Ruthenium complexes with polydentate chelating agents ("secondary ligands") may be considered as prodrugs against different tumors. The role of the secondary ligand is to stabilize the ruthenium (III) cation so that the metal is not delivered to the tissues during its transportation in the body and the whole complex remains unchanged. In this way, a drastic lowering of the host toxicity produced by these potential drugs have been observed. Once the complex reaches the hypoxic areas proper of tumor tissues, the metal ion is reduced "in situ", this feature promoting the accumulation of ruthenium in tumor masses[10,11]. As published[10], a PDTA-Ru(III) complex accumulates in tumor tissues (female Swiss mice bearing EMT-6 solid sarcoma) in higher proportion (TCI = 2.13) than other ruthenium compounds, i.e., TCI = 1.70 for $Ru(en)_2Cl_2$ (TCI = tumor concentration index : % of inyected doses per g of tumor/ % inyected doses per g of total weight). At the same time, the rate drug dose/g of tissue of PDTA-Ru drug found in liver and kidneys is much lower (2.30 and 5.42 %) than in the $Ru(en)_2Cl_2$ compound (5.76 and 11.15 %). It has been proposed that the "primary ligands" (i.e., chlorine atoms or sulfate, nitrate, carbonate, oxalate, malonate anions) are replaced by nitrogen or oxygen atoms from the nucleobases of cellular DNA, purine and pyrimidine bases, if the DNA is the macromolecular target of these complexes[5]. Research in progress evidences that this type of metal drugs interferes in the growing process of human mammary carcinomas[12].

In the present paper, we characterize and isolate in crystalline form a new compound of Ru(III) with PDTA. The study of the properties employs the use of chemical analyses, potentiometry, vibrational spectroscopy and X-ray diffraction structural studies. The obtained data made it possible to reach conclusions about the strength of the bonds formed by ruthenium with nitrogen and oxygen atoms of the chelating ligand and also with the chlorine atoms directly bonded to the metal ion. Finally, the activity of the new potential drug against different experimental and human tumors has been tested "in vivo".

### Materials and Methods

### *Chemicals*

Hydrated ruthenium (III) chloride was used as provided by Johnson Matthey. All chemicals obtained from commercial suppliers were used without further purification. Water of Millipore quality was used in all solutions.

### *Elemental analyses*

Elemental microanalyses were performed at the Analytical Chemistry Department of the Sevilla University. Hydration water was determined by Karl-Fischer titrimetry. Metal content was determined by atomic absorption spectroscopy using a Perkin Elmer 2380 model, at 10 mA and $\lambda$ of 349.9 nm.

### *Titrimetry*

Potentiometric measurements were carried out using a Crison MicroTT 2022 titrimeter, provided with autoburette Microbur 2030. Aqueous solutions of the complex (30-60 mg/100 mL) were titrated against NaOH 29.5 mM solution. Electrical conductimetry of the same solutions were performed using a Crison 525 conductimeter.

### *Spectroscopic and magnetic studies*

Infrared spectra were recorded on Perkin Elmer 883 (4000-400 $cm^{-1}$ range) and FT-IR 16PC (400 - 200 $cm^{-1}$ interval) instruments, respectively. The spectra in the 4000-400 $cm^{-1}$ range were taken as KBr discs, while far-infrared spectra were measured as Nujol mulls supported between polyethylene sheets. Electronic spectra were taken with a Jasco V-550 spectrometer while magnetic measurements were carried out in a Faraday type equipment equipped with helium cryostat at temperatures between 70 and 300K. The balance was calibrated with standard $Hg[Co(SCN)_4]$. Pascal Tables were used for diamagnetic corrections.

### *Antitumor essays*

The cytotoxic effect of the ruthenium compound has been evaluated against Ehrlich ascitic tumor (EAT) and the murine or human carcinomas: limphocytic L1210 and P388 leukemias, M5076 intraperitoneal implanted sarcoma, and MX-1 human transplanted mammary sarcoma. Most of the experiments were performed at NIH, Bethesda, USA and ONC, Madrid, Spain.

### *X-ray diffraction*

A brown, irregular prismatic crystal, dimensions 0.1 x 0.2 x 0.3 mm was mounted in a Stoe-Siemens AED 2 diffractometer with MoKa radiation ($\lambda$ = 0.71073 Å). A total of 5898 independent reflections were measured in the range $-26 \leq h \leq 26$, $-10 \leq k \leq 10$, $-20 \leq l \leq 20$, $2\Theta < 60°$, 4947 with $F > 4\sigma(F)$ retained for structure solution and refinement. Data were corrected for Lorentz and polarisation and absorption (empirically, $\chi$ scans, transmission range, 0.623-0.781). Ruthenium and chlorine atoms were located from a Patterson map, other atoms from successive $\Delta F$ maps. The structure was refined anisotropically for non-H atoms, minimizing $\Sigma w(F_o-F_c)^2$ with $w^{-1} = \sigma^2(F) + 0.001 F^2$.

**Table 1.** Atomic coordinates (Ru and Cl, x$10^5$; other, x$10^4$) and equivalent isotropic displacement coefficients ($Å^2$x$10^3$)

| | x | y | z | U(eq) |
|---|---|---|---|---|
| Ru | 7521(3) | 14272(3) | 0 | 24.87(6) |
| Cl(1) | 4013(6) | 28254(18) | -13788(8) | 43.1(3) |
| Cl(2) | 17342(6) | 149(17) | -7310(8) | 43.4(3) |
| C(1) | 596(2) | 1449(5) | 1976(3) | 29(1) |
| C(2) | -167(2) | 1446(5) | 1634(3) | 28(1) |
| C(3) | -665(2) | 2279(7) | 2352(3) | 41(1) |
| N(1) | 1097(2) | 555(4) | 1311(2) | 26(1) |
| C(11) | 1840(2) | 1285(6) | 1427(3) | 34(1) |
| C(12) | 1901(2) | 3070(6) | 932(3) | 31(1) |
| O(11) | 1415(2) | 3472(4) | 366(2) | 33(1) |
| O(12) | 2427(2) | 4049(5) | 1103(2) | 44(1) |
| C(13) | 1068(2) | -1417(5) | 1375(3) | 30(1) |
| C(14) | 1310(2) | -2223(6) | 2271(3) | 33(1) |
| O(13) | 1591(2) | -1413(4) | 2898(2) | 47(1) |
| O(14) | 1179(2) | -3953(4) | 2293(2) | 43(1) |
| N(2) | -193(2) | 2339(4) | 698(2) | 25(1) |
| C(21) | -801(2) | 1568(5) | 155(3) | 30(1) |
| C(22) | -592(2) | -250(5) | -232(3) | 29(1) |
| O(21) | 77(1) | -636(4) | -277(2) | 32(1) |
| O(22) | -1063(2) | -1297(4) | -504(2) | 42(1) |
| C(23) | -201(2) | 4323(5) | 717(3) | 30(1) |
| C(24) | -885(2) | 5260(5) | 1022(3) | 31(1) |
| O(23) | -1488(2) | 4717(5) | 890(3) | 57(1) |
| O(24) | -733(2) | 6803(4) | 1408(3) | 40(1) |
| O(1W) | 1820(2) | 1485(5) | -2806(3) | 52(1) |
| O(2W) | 2592(2) | 6017(6) | -334(3) | 63(1) |
| O(3W) | 1919(2) | 4882(6) | -1674(3) | 62(1) |
| O(4W) | -1542(3) | -4486(7) | -1224(4) | 86(2) |

* Equivalent isotropic U defined as one third of the trace of the orthogonalized $U_{ij}$ tensor

Hydrogen atoms attached to carbon were idealized whereas those attached to oxygen were located in ΔF maps and refined with fixed O-H distances (0.85 Å). Final residues were R = 0.033, $R_w$ = 0.033, S = 1.02, -1.39 < ΔF < 0.91 e.$Å^{-3}$, shift/σ below 0.001 in the last cycle. Final atomic coordinates are included in Table 1, hydrogen coordinates, anisotropic thermal parameters are included in the supplementary material. Tables of structure factors are available from the authors on request.

***Synthesis of the compound [RuLCl$_2$]H.4H$_2$O (L = PDTA)***

A solution of $RuCl_3.xH_2O$ (0.50 g, 2.0 mmol) in 200 ml of HCl 0.1 M is introduced in a 500 ml round-bottom flask and 0.62 g of solid PDTA is carefully added on the ruthenium solution while stirring. The mixture is magnetically stirred for about 10 minutes. The flask is air-tight sealed and heated for about 12 hours at 130 ºC in an electric oven. Along this time, all the ligand is dissolved and the initial dark-red colour of the mixture changes to pale-purple colour. After cooling at room temperature, the solution is filtered and slowly evaporated in water-bath at 70ºC until small volume (i.e, 10 ml). Red-brown prismatic crystals are recovered after filtration from the mother liquor. Anal. Calcd. for $C_{11}H_{24}O_{12}N_2Cl_2Ru$: C, 24.05; H, 4.59; N, 5.10; Cl 12.9; Ru, 18.4; $M^+$ = 548.3. Found: C, 24.2; H, 4.29; N, 5.19; Cl, 12.9; Ru, 18.3; $M^+$ = 549.

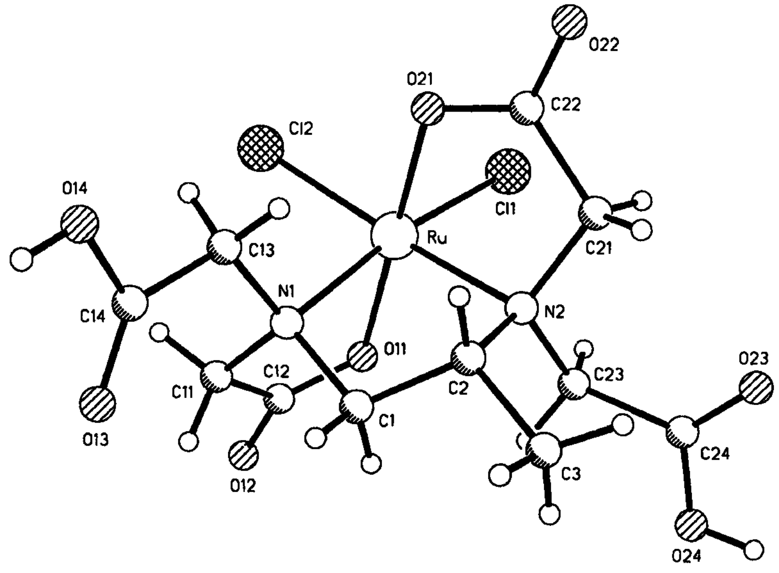

Figure 2: Molecular structure of $[RuLCl_2]H.H_2O$

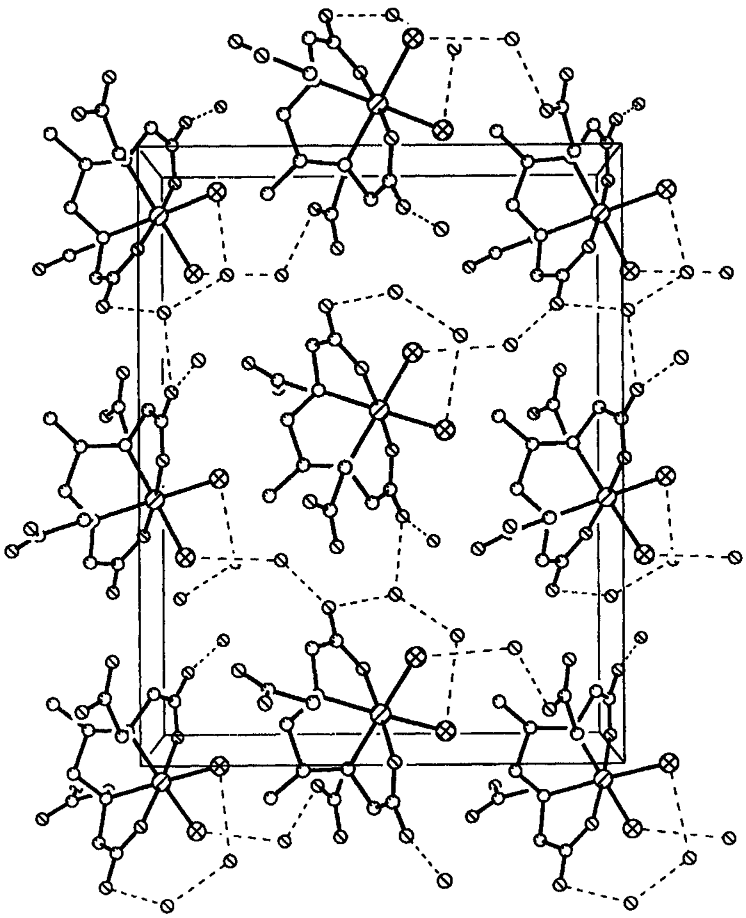

Figure 3: Stereoview of the unit cell packing diagram for $[RuLCl_2]H.H_2O$

### Results and Discussion

### *Isolation of the ruthenium (III) complex*

The synthesis of the title complex is performed in a molar metal / ligand ratio of 1/1 and the reaction takes places in a sealed flask at 130ºC. In the conditions explained in the experimental section, a yield of 72.3 % is obtained. The solid is extremely soluble in water and stable to air at room temperature.

### *Crystallographic studies*

A view of the anion dichloro(propylendiaminetetraacetato) ruthenate(III) is shown in Figure 2. Crystal and refinement data for $[RuLCl_2]H.4H_2O$ are summarized in Table 2. Bond distances and angles are indicated in Table 3. The metal atom is octahedrally coordinated by two chlorine atoms, placed in *cis* positions and the organic ligand occupying the remaining four vertexes of the octahedron via the two nitrogen atoms (*trans* to chlorines) and two carboxylate groups, closing three five-membered chelate rings. The non-coordinated carboxylic acid groups are clearly protonated, as can be seen from the corresponding C-O distances and the hydrogen bond scheme, the attached hydrogen atoms readily located in difference Fourier maps. The structure of this complex ion is similar to the analogous with EDTA and very close to the one with DCTA[6,9], a difference being that the distances Ru-Cl are more similar to each other in the PDTA compound. As in the DCTA complex, Ru-Cl bond distances [2.361(1) Å and 2.362(1)Å] are much longer than the reported in literature for similar compounds[4,13], this fact contributing to enhance the labile character of the halide ligands. The equal distances found for the Ru-O bonds indicate the absence of tetragonal distortion. A noteworthy difference between the PDTA and DCTA complexes of ruthenium is found considering the magnitude of the angles around the ruthenium atom; these angles are not far away from ideal octahedral geometry in the PDTA complex, with the logical closing of the N-Ru-O angles inside the chelate rings and the opening of their supplementaries.

**Table 2.** Crystallographic data for $[RuLCl_2]\ H.4H_2O$

| | |
|---|---|
| Formula | $C_{11}H_{24}N_2O_{12}Cl_2Ru$ |
| Molecular weight | 548.3 |
| Crystal system | orthorhombic |
| a(Å) | 18.571(3) |
| b(Å) | 7.464(1) |
| c(Å) | 14.617(3) |
| V(Å$^3$) | 2026.2(8) |
| Space group | $Pna2_1$ |
| Z | 4 |
| $D_{calc}$ (g cm$^{-3}$) | 1.797 |
| F(000) | 1112 |
| $\lambda$ (MoK$\alpha$) (Å) | 0.71073 |
| $\mu$ (MoK$\alpha$) (mm$^{-1}$) | 1.099 |
| Reflections measured | 5898 |
| R (= $R_w$) | 0.033 |

All water molecules and the carboxylic acid groups act as donors in hydrogen bonding (see Table 3), the acceptors being water molecules, carboxylate groups and chlorine atoms. A view of some of these hydrogen bonds is displayed in Figure 3.

The oxidation state of ruthenium is +3, as shown by magnetic measurements, so an extra proton must be attached to any of the water molecules in order to balance the charge of the complex anion. Nevertheless, difference maps and the hydrogen bonds displayed in Table 3 let us to identify clearly only two hydrogen atoms per water oxygen, the remaining proton being probably disordered among water molecules.

**Table 3.** Interatomic distances (Å) and bond angles (°)

| | |
|---|---|
| Ru-Cl(1) | 2.361 (1) |
| Ru-Cl(2) | 2.362 (1) |
| Ru-N(1) | 2.122 (3) |
| Ru-O(11) | 2.033 (3) |
| Ru-N(2) | 2.142 (3) |
| Ru-O(21) | 2.026 (3) |
| C(1)-C(2) | 1.503 (5) |
| C(1)-N(1) | 1.502 (5) |
| C(2)-C(3) | 1.531 (6) |
| C(2)-N(2) | 1.523 (5) |
| N(1)-C(11) | 1.493 (5) |
| N(1)-C(13) | 1.476 (5) |
| C(11)-C(12) | 1.521 (6) |
| C(12)-O(11) | 1.260 (5) |
| C(12)-O(12) | 1.245 (5) |
| C(13)-C(14) | 1.510 (6) |
| C(14)-O(13) | 1.215 (5) |
| C(14)-O(14) | 1.314 (5) |
| N(2)-C(21) | 1.496 (5) |
| N(2)-C(23) | 1.481 (5) |
| C(21)-C(22) | 1.521 (5) |
| C(22)-O(21) | 1.276 (4) |
| C(22)-O(22) | 1.239 (5) |
| C(23)-C(24) | 1.518 (5) |
| C(24)-O(23) | 1.207 (5) |
| C(24)-O(24) | 1.313 (5) |

*Hydrogen bonds:*

| | |
|---|---|
| O(14)→O(4W)$^{i}$ | 2.552(6) |
| O(24)→O(1W)$^{ii}$ | 2.650(5) |
| O(1W)→Cl(2) | 3.230(4) |
| O(1W)→O(12)$^{iii}$ | 2.793(5) |
| O(2W)→O(12) | 2.582(6) |
| O(2W)→O(22)$^{iv}$ | 2.520(5) |
| O(3W)→O(2W) | 2.472(7) |
| O(3W)→Cl(1) | 3.239(4) |
| O(4W)→O(22) | 2.751(6) |
| O(4W)→Cl(2)$^{v}$ | 3.305(5) |

$^{i}$ $-x,-y-1,z+\frac{1}{2}$
$^{ii}$ $-x,-y+1,z+\frac{1}{2}$
$^{iii}$ $-x+\frac{1}{2},y-\frac{1}{2},z-\frac{1}{2}$
$^{iv}$ $x+\frac{1}{2},-y+\frac{1}{2},z$
$^{v}$ $x-\frac{1}{2},-y+\frac{1}{2},z$

| | |
|---|---|
| Cl(1)-Ru-Cl(2) | 91.38(4) |
| Cl(1)-Ru-N(1) | 171.61(9) |
| Cl(2)-Ru-N(1) | 92.20(9) |
| Cl(1)-Ru-O(11) | 93.44(9) |
| Cl(2)-Ru-O(11) | 89.22(9) |
| N(1)-Ru-O(11) | 79.0(1) |
| Cl(1)-Ru-N(2) | 92.30(9) |
| Cl(2)-Ru-N(2) | 172.03(8) |
| N(1)-Ru-N(2) | 85.1(1) |
| O(11)-Ru-N(2) | 97.6(1) |
| Cl(1)-Ru-O(21) | 89.69(8) |
| Cl(2)-Ru-O(21) | 92.76(8) |
| N(1)-Ru-O(21) | 97.7(1) |
| O(11)-Ru-O(21) | 176.3(1) |
| N(2)-Ru-O(21) | 80.2(1) |
| C(2)-C(1)-N(1) | 111.6(3) |
| C(1)-C(2)-C(3) | 110.0(3) |
| C(1)-C(2)-N(2) | 109.2(3) |
| C(3)-C(2)-N(2) | 114.7(3) |
| Ru-N(1)-C(1) | 105.1(2) |
| Ru-N(1)-C(11) | 105.7(2) |
| C(1)-N(1)-C(11) | 109.6(3) |
| Ru-N(1)-C(13) | 110.6(2) |
| C(1)-N(1)-C(13) | 112.3(3) |
| C(11)-N(1)-C(13) | 113.0(3) |
| N(1)-C(11)-C(12) | 109.5(3) |
| C(11)-C(12)-O(11) | 117.9(3) |
| C(11)-C(12)-O(12) | 118.5(4) |
| O(11)-C(12)-O(12) | 123.6(4) |
| Ru-O(11)-C(12) | 115.3(3) |
| N(1)-C(13)-C(14) | 116.2(3) |
| C(13)-C(14)-O(13) | 125.7(4) |
| C(13)-C(14)-O(14) | 110.9(4) |
| O(13)-C(14)-O(14) | 123.4(4) |
| Ru-N(2)-C(2) | 105.2(2) |
| Ru-N(2)-C(21) | 104.1(2) |
| C(2)-N(2)-C(21) | 109.5(3) |
| Ru-N(2)-C(23) | 109.5(2) |
| C(2)-N(2)-C(23) | 114.9(3) |
| C(21)-N(2)-C(23) | 112.8(3) |
| N(2)-C(21)-C(22) | 110.3(3) |
| C(21)-C(22)-O(21) | 118.0(3) |
| C(21)-C(22)-O(22) | 120.1(3) |
| O(21)-C(22)-O(22) | 121.9(4) |
| Ru-O(21)-C(22) | 114.8(2) |
| N(2)-C(23)-C(24) | 118.3(3) |
| C(23)-C(24)-O(23) | 125.1(4) |
| C(23)-C(24)-O(24) | 110.6(3) |
| O(23)-C(24)-O(24) | 124.2(4) |

**Antitumor activity**

The antitumor activity of the synthesized ruthenium complex has been evaluated against the murine and human tumors mentioned in the experimental part of this paper. The results obtained are reported in Table 5. The complex exhibits a noteworthy activity against Ehrlich ascitic tumor, with a maximum T/C value of 460 correspopnding to a dose of 120 mg/Kg. The ruthenium drug presents an important antitumor action against the murine leukemias L1210 and P388, it being the optimum dose the same as in EAT case. The most important finding is the marked cytotoxic effect shown by the ruthenium complex against the MX-1 human mammary tumor, even larger than in the case of DCTA-Ru compound[9]. Cures are complete in the EAT case with a long term survivors of 8/8 and appreciable in the L1210 and P388 leukemias (LTS of 4/8 and 3/8, respectively). The complex is exceptionally well tolerated and shows an absolute lack of nephrotoxicity and liver toxicity at all given doses. Moreover, myelosuppression is not observed.

**Table 5**. Antitumor activity (T/C, %) of the complex $[RuLCl_2]H.4H_2O$

| | Tumors | | | | |
|---|---|---|---|---|---|
| doses (mg/Kg) | EAT | L1210 | P388 | MX-1 | M5076 |
| 10 | 180 | 110 | | | |
| 25 | 360 | 130 | 125 | | |
| 60 | 410 | 210 | 150 | 13 | 125 |
| 120 | 460 | 190 | 160 | 10 | 130 |
| 250 | 130 | 110 | 125 | | 100 |

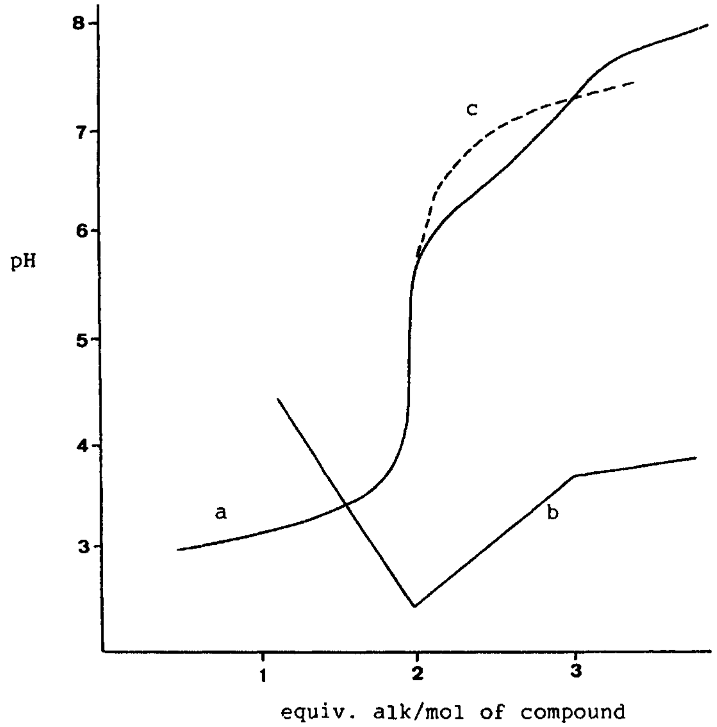

Figure 4: Potentiometric study of the complex $[RuLCl_2]H.4H_2O$

Molar conductance (298K) changes from $\Lambda$ = 174 S $cm^2$ (1 g-equiv. alkali) to $\Lambda$ = 151 S $cm^2$ (2 g-equiv. alkali)

### Potentiometric Study

Figure 4a corresponds to the potentiometric titration of a freshly prepared aqueous solution of $[RuLCl_2]$ $H.4H_2O$. As can be seen, a sharp increase of pH is registered for the consumption of 2 g-equiv. of alkali. The inflection point appears at pH 4.9. Moreover, a sudden change in the electrical conductivity of the solution is observed at this point (Figure 4b), this fact demonstrating the simultaneous neutralization of two free -COOH groups of the same strength. These results evidence a tetradentate character for the PDTA, that contains two coordinated carboxylate groups and two free carboxylic groups. The additional smooth inflection of the curve observed at 3 g-equiv. of alkali may corresponds to the presence of a third weak acid hydrogen atom somehow externally bonded to the complex. The pK value calculated from the curve is of 1.92, according to previous measurements on similar compounds (14).

All these facts have been checked by titration of the chlorine atoms present in the substance. Dissolution of the compound in water leads to the slow substitution of one chlorine atom from the inner sphere of the complex. If a freshly prepared solution is titrated with $AgNO_3$, 0.60 g-equiv. of $Cl^-$ are titrated whereas in a solution left to stand for 36 h, 1.43 g-equiv. of $Cl^-$ were found. If the position of the chlorine atom is taken by water, the substituted $Cl^-$ must neutralize the external $H^+$. This fact has been confirmed by a new potentiometry, as observed in Figure 4c, in which the second inflection has disappeared. Consequently, the coordinated PDTA contains two free carboxylic groups even after the replacement of the chlorine atom.

### Infrared spectroscopy

The isolated complex shows bands in the infrared spectra indicating the presence of both, non-coordinated -COOH groups (1720 $cm^{-1}$) and coordinated -$COO^-$ groups (1610 $cm^{-1}$). Additional important vibrational modes are registered at 530 $cm^{-1}$(Ru-N bond) and by a split peak at 330 $cm^{-1}$ and 290 $cm^{-1}$, this fact indicating the presence of two chlorine atoms bonded to ruthenium in *cis* positions. Other characteristic peaks of the complex appear in Table 4.

**Table 4.** IR data ($cm^{-1}$) for the complex $[RuLCl_2]H.4H_2O$

| COOH (C=O) | $COO^-$ | $CH_2$ (C-H) | $H_2O$ hyd | Ru-N | Ru-Cl |
|---|---|---|---|---|---|
| 1720 $\nu_s$ | 1610 $\nu_s$ | 2920 | 3510 $\nu_s$ | 530 | 330 |
| 840 | 1380 $\nu_{as}$ | 2960 | 1630 $\delta$ | | 295 |

$\nu_s$ and $\nu_{as}$: symmetric and asymmetric stretching vibrations;
$\delta$: bending vibration.

### Electronic and magnetic studies

The electronic spectra of aqueous solutions of the complex show three 'bands at 22830 $cm^{-1}$ , 28570 $cm^{-1}$ and 34480 $cm^{-1}$. Extinction coefficients are larger than those calculated for Ru-EDTA analogs. The registered absorptions may be attributed to the transitions $^2A_{2g}\leftarrow{}^2T_{2g}$, $^2T_{1g}\leftarrow{}^2T_{2g}$ and $^2E_g$ $^{-2}T_{2g}$.

The magnetic behaviour of the substance is characteristic of a Curie-Weiss paramagnet in the temperature range studied (80-298K). The magnetic moment changes from 1.76 $\mu_B$ (298K) to 1.89 $\mu_B$ (80K), which is as expected for one unpaired electron spin. These and other data (X-ray Photoelectron Spectroscopy) are indicative of the +3 oxidation state for ruthenium, with a low-spin $4d^5$ configuration.

### Acknowledgements

We thank the financial support from the Commission of the European Communities (program HCM, contract no. ERBCHRXCT 920016) and from CICYT, Spain (roject SAF-0467-94). We are grateful to Johnson Matthey for a generous loan of platinum metals.

### References

1. González-García, S., González-Vílchez, F., *Anal. Quím*., 1970, **66**, 845.
2. Taqui Khan, M.M., Kumar, A., Shirin, Z., *J. Chem. Res. Synop*.,1986, 130.
3. Bajaj, H.C., van Eldik, R.., *Inorg. Chem*., 1988, **27**, 4052.
4. Taqui Khan, M.M., Chatterjee, D., Merchant, R.R., Paul, P., Abdi, S.H.R., Srinivas, D., Siddiqui, M.R.H., Moiz, M.A., Bhadbhade, M.M., Venkatasubramanian,K., *Inorg. Chem*., 1992, **31**, 2711.
5. Clarke, M.J., in: *Platinum, Gold and Other Metal Chemotherapeutic Agents* : Lippard, S.J. (ed.) Washington: ACS Symp. Ser., 1983, pp. 335-352.
6. Vilaplana, R., Basallote, M.G., Ruiz-Valero, C., Gutierrez-Puebla, E., González-Vílchez, F., *J. Chem. Soc., Chem. Commun*., 1991, 100.
7. Keppler, B.K., Friesen, C., Vongerichten, H., Vogel, E., in: *Metal Complexes in Cancer Chemotherapy* : Keppler, B.K. (ed.) Weinheim:VCH, 1993, pp. 297-324.
8. Clarke, M.J., in: *Metal Complexes in Cancer Chemotherapy* : Keppler, B.K. (ed.) Weinheim: VCH, 1993, pp. 129-156.
9. Vilaplana, R., González-Vílchez, F., Gutierrez-Puebla, E., Ruiz-Valero, C., *Inorg. Chim. Acta* , 1994, **224**, 15.
10. González-Vílchez, F., Vilaplana, R., in: *Trends in Cancer Research* : Barbera, E. (ed.) Vizcaya: UPV, 1986, pp. 194-198.
11. Mestroni, G., Alessio, E., Sava, G., Pacor, S., Coluccia, M. Boccarelli, A., *Metal -Based Drugs* , 1994, **1**, 43.
12. Moreno, J., García-Erdugo, G., González-Vílchez, F., Vilaplana, R., unpublished work.
13. Bats, J.W., Pandey, K.K., Roesky, H.W., *J. Chem. Soc., Dalton Trans*., 1984, 2081.
14. Shimizu, K., Matsubara, T., Sato, G.P., *Bull. Soc. Chem. Jpn.,* 1974, **47**, 1651, and references therein.

**Received: May 2, 1995 - Accepted: May 18, 1995 - Received camera-ready revised version: May 22, 1995**

